# Non-selective excitatory feedback and precise spike timing produce selective relative inhibition

**DOI:** 10.1186/1471-2202-15-S1-P15

**Published:** 2014-07-21

**Authors:** Biao Han, Rufin VanRullen

**Affiliations:** 1 Centre de Recherche Cerveau et Cognition, Université de Toulouse, Toulouse, 31062, France; 2 CNRS, UMR 5549, Faculté de Médecine de Purpan, CHU Purpan, Toulouse Cedex, 31052, France

## 

The essence of feedback is key to understand the brain. However, inconsistencies exist between different observation levels. In neurophysiology, feedback connections are considered as excitatory. On the other hand, several electrophysiology and neuroimaging studies suggest that feedback connections are inhibitory. Additionally, many computational theories (such as predictive coding) assume that feedback produces selective inhibition of a subset of inputs; this often requires dedicated feedback circuitry with inhibitory connections that mirror the feed-forward excitatory connectivity. Here, we show that relative inhibition of a selected subset of inputs can be produced by strictly excitatory and non-selective feedback connections, together with precise spike times.

**Figure 1:**
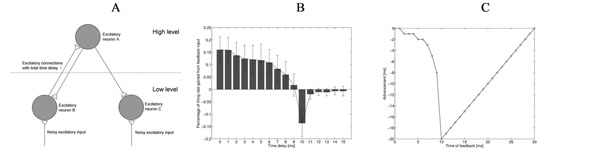
A. The neural network model B. Results of parameter exploring with different conduction delays τ. Positive values indicate selective inhibition of neuron B relative to neuron C. C. Difference in advancement time (a measure of feedback excitation) depending on the time of feedback spike relative to previous output spike.

As shown in Figure 1.A, we designed a simplified neural network with one high-level excitatory neuron and two low-level excitatory neurons. Each neuron, modelled by a leaky integrate-and-fire (LIF) unit or an exponential integrate-and-fire (EIF) unit, represents a distinct neuron population. The two low-level neurons have the same constant input (with additive noise). The high-level neuron (A) is “selective” for inputs from low-level neuron (B) (strongly weighted feed-forward connection) while discounting inputs from low-level neuron (C) (weakly weighted connection). In turn, the high-level neuron (A) sends equivalent (non-selective) excitatory feedback to both low-level neurons. The conduction time of both feedforward and feedback connections is denoted by τ.

We hypothesized that precise spike timing relations between the 3 neurons would result in feedback inhibition of low-level neuron (B) relative to low-level neuron (C). That is, feedback selectively inhibits the strongly-weighted inputs of the high-level neuron. To quantify this effect, we measured the increase of firing rate of neuron (C) relative to (B), for various values of the following parameters: neuron model types (LIF or EIF), membrane time constant, intensity of the constant input to low-level neurons, additive noise range to low-level neurons, and conduction delay τ. Results for parameter τ are shown in Figure 1.B as an example. All results indicate that it is possible to generate a relative inhibitory effect with biologically plausible parameters.

The principle behind this effect can be simply summarized as a sensitivity difference to incoming (feedback) spikes at different stages of the leaky integrate-and-fire process (as observed experimentally in neuronal recordings [1]). As shown in Figure 1.C, the effect on a low-level neuron of receiving a feedback spike changes dramatically according to the feedback time relative to the preceding output spike from the low-level neuron. When the time delay between the output spike and the feedback spike is short (which is more likely for low-level neuron B due to its strong connectivity to the higher-level neuron), excitatory feedback has relatively little influence on the low-level neuron’s firing, because it arrives at a time when the neuron is too far from threshold; the leak will then reduce any contribution of the feedback spike before threshold is reached. This, however, is less likely to occur for neuron C, whose output spikes are decorrelated from feedback spikes. As a result, relative feedback inhibition emerges selectively for the strongly-weighted low-level neurons (as postulated by predictive coding theory).
